# Randomized Controlled Trials in Lung, Gastrointestinal, and Breast Cancers: An Overview of Global Research Activity

**DOI:** 10.3390/curroncol29040207

**Published:** 2022-04-07

**Authors:** J. Connor Wells, Adam Fundytus, Shubham Sharma, Wilma M. Hopman, Joseph C. Del Paggio, Bishal Gyawali, Deborah Mukherji, Nazik Hammad, C. S. Pramesh, Ajay Aggarwal, Richard Sullivan, Christopher M. Booth

**Affiliations:** 1 Division of Cancer Care and Epidemiology, Queen’s University Cancer Research Institute, Kingston, ON K7L 3N6, Canada; connor.wells@bccancer.bc.ca (J.C.W.); adam.fundytus@queensu.ca (A.F.); shsharma@qmed.ca (S.S.); bishal.gyawali@kingstonhsc.ca (B.G.); 2 Department of Oncology, Queen’s University, Kingston, ON K7L 3N6, Canada; nazik.hammad@kingstonhsc.ca; 3 Department of Public Health Sciences, Queen’s University, Kingston, ON K7L 3N6, Canada; wilma.hopman@kingstonhsc.ca; 4 Department of Oncology, Northern Ontario School of Medicine, Thunder Bay, ON P7B 5E1, Canada; jdelpagg@lakeheadu.ca; 5 Department of Internal Medicine, Faculty of Medicine, American University of Beirut Medical Center, Beirut 1107 2020, Lebanon; dm25@aub.edu.lb; 6 Tata Memorial Centre, Homi Bhabha National Institute, Mumbai 400012, India; prameshcs@tmc.gov.in; 7 Institute of Cancer Policy, King’s College London, London SE1 9RT, UK; ajay.aggarwal@kcl.ac.uk (A.A.); richard.sullivan@kcl.ac.uk (R.S.); 8 Department of Health Services Research and Policy, London School of Hygiene and Tropical Medicine, London WC1E 7HT, UK

**Keywords:** randomized controlled trial, gastrointestinal, lung, breast, cancer, design, outcomes

## Abstract

Background: In this study, we compared and contrasted design characteristics, results, and publications of randomized controlled trials (RCTs) in gastrointestinal (GI), lung, and breast cancer. Methods: A PUBMED search identified phase III RCTs of anticancer therapy in GI, lung, and breast cancer published globally during the period 2014–2017. Descriptive statistics, chi-square tests, and the Kruskal–Wallis test were used to compare RCT design, results, and output across the cancer sites. Results: A total of 352 RCTs were conducted on GI (36%), lung (29%), and breast (35%) cancer. Surrogate endpoints were used in 55% of trials; this was most common in breast trials (72%) compared to GI (47%) and lung trials (43%, *p* < 0.001). Breast trials more often met their primary endpoint (54%) than GI (41%) and lung trials (41%) (*p* = 0.024). When graded with the ESMO-MCBS, lung cancer trials (50%, 15/30) were more likely to meet the threshold for substantial benefit. GI trials were published in journals with a substantially lower impact factor (IF; median IF 13) than lung (median IF 21) and breast cancer trials (median IF 21) (*p* = 0.038). Conclusions: Important differences in RCT design and output exist between the three major cancer sites. Use of surrogate endpoints and the magnitude of benefit associated with new treatments vary substantially across cancer sites.

## 1. Introduction

Cancers of the gastrointestinal (GI) tract, lung, and breast account for nearly half of the global cancer burden and over 60% of annual cancer deaths [1]. Mortality in these cancers has been declining, and while these improved population-level outcomes likely reflect earlier diagnosis and changes in lifestyle (i.e., smoking), improvements in cancer treatment also play a role in some of the observed gains in population health [2,3,4,5,6]. These important advances in treatment are based on the results of phase III randomized controlled trials (RCTs).

The RCT remains the cornerstone of evidence-based cancer care. However, in recent years, there have been concerns that RCT design may be compromised through increased use of unvalidated surrogate endpoints for overall survival (OS), inappropriate control arms, and the inherent tension between statistical significance and clinical significance [7,8,9,10,11]. Additionally, in the last decade, the price of cancer therapies has increased at a remarkable rate [12]. These costs pose considerable challenges to patients and healthcare systems and are compounded by the marginal effect size associated with many new cancer therapies [13,14].

To understand the global cancer ecosystem, we recently reported the results of a study that describes all cancer RCTs published globally during the period 2014–2017 [15]. Key findings from this global overview include the identification of a mismatch of RCTs and the global cancer burden (i.e., many cancers are under-represented in RCTs), a paucity of trials in surgery and radiotherapy, frequent use of surrogate outcomes, and the finding that only a minority of new cancer therapies provide substantial clinical benefit. There has also been a striking shift in the funding of RCTs, with 71% of all published trials now funded by industry [7,8]. Among the 694 RCTs published globally during this time period, more than 50% tested new cancer therapies for patients with breast, lung, and gastrointestinal cancers. Given the public health burden of these three diseases, and the substantial proportion of global cancer research resources devoted to their study, we undertook the current study to compare and contrast design characteristics, results, and publications of RCTs for these cancers. Insights from clinical trials across these three major cancer sites can inform the design of future studies to maximize the patient-level and societal benefits of clinical cancer research.

## 2. Methods

### 2.1. Study Design and Search Strategy

This study is a secondary analysis of a recently published retrospective cohort study of global oncology RCTs published between 2014 and 2017. The original study design and cohort generation are described elsewhere [15]. A structured PUBMED literature search identified phase III RCTs evaluating cancer therapy (including systemic, radiation, and surgery). Studies were included if they were English-language reports with a phase III clinical design, evaluated any malignancy, and evaluated a cancer-directed therapy. Studies were excluded if they reported only subset/pooled analysis or interim analysis or assessed screening/prevention. Randomized phase II trials were also excluded. In case the trial had been reported more than once, the paper where the primary endpoints are described, or the more recent publication, was included. Supportive therapies, such as antiemetics, and integrative medicine (i.e., vitamins) were excluded.

### 2.2. Data Abstraction and Classification

Data collection was performed independently by two authors (J.C.W. and S.S.) using a standardized collection template. Variables included authorship, funding, study design, results, and journal of publication; the senior author (C.M.B.) performed random data checks to ensure abstraction was of high quality. At the completion of data collection, a further 30 studies were randomly evaluated for concordance; only 11/1020 (1%) variables were discordant with the original assessment. Another author (J.D.P.) derived European Society of Medical Oncology-Magnitude of Clinical Benefit Results Scale (ESMO-MCBS version 1.1) grades for all superiority trials of systemic therapy with a statistically significant difference in favor of the experimental arm [16].

### 2.3. Outcomes and Statistical Analysis

Statistical analysis was conducted using IBM SPSS version 26.0 for Windows (Armonk, NY, USA, 2019). Descriptive results (frequencies, percentages, medians, and quartiles) were generated for the full study cohort and by disease site. Comparisons were then performed between GI, lung, and breast RCTs using the Pearson chi-square or Fisher’s exact test, and the Mann–Whitney U or the Kruskal–Wallis test as appropriate. The funding source is identified by explicit statements in the manuscript or in the acknowledgement section. Studies were classified into country of origin based on the institutional affiliation of the first author; country of origin was used to further divide studies into low-middle/upper-middle-income countries (collectively referred to as LMICs) or high-income countries (HICs) [17]. The journal impact factor (IF) was compared using the IF from 2016 as reported by the Journal Citation Reports Impact Factor [18]. For ESMO-MCBS analyses, grades of A and B (in the curative setting) and 5 and 4 (in the palliative setting) were considered to be of “substantial” benefit [16,19]. Values of *p* less than 0.05 were considered significant; no adjustments were made for multiple comparisons.

## 3. Results

### 3.1. Results of the Search Strategy

The search strategy identified 2275 publications, 694 of which represented phase III RCTs of anticancer therapy. We identified 352 RCTs conducted on GI (*n* = 127, 36%), lung (*n* = 104, 29%), and breast (*n* = 121, 35%) cancer; these comprised the study cohort. Of the 127 GI trials, 58 (46%) were colorectal, 38 (30%) were gastric/esophageal, 14 (11%) were pancreas/biliary, 14 (11%) were hepatocellular carcinoma, and 3 (2%) were other. Disease subtype was not captured for lung or breast cancer.

### 3.2. Design Characteristics of RCTs

Approximately 50% of trials were led by one of four countries: the United States (67 trials, 19%), Japan (50 trials, 14%), the United Kingdom (32 trials, 9%), and France (30 trials, 9%). The majority of trials were funded by industry (75%, 263/352); GI trials (67%, 85/127) were less likely than lung (79%, 82/104) and breast trials (79%, 96/121) to be funded by industry (*p* = 0.037).

The design characteristics of trials by disease site are listed in Table 1. A total of 89% and 11% of trials were led by HICs and LMICs, respectively. A larger proportion of lung cancer trials were led by LMICs (19%, 20/104) than GI (9%, 11/127) or breast trials (5%, 6/121) (*p* = 0.002). Approximately two thirds of trials were conducted in the palliative setting (63%, 223/352). Breast trials were more likely to be in the curative context (58%, 70/121) than GI (35%, 45/127) or lung trials (13%, 14/104) (*p* < 0.001). Conversely, lung cancer trials (87% 90/104) were more likely to be conducted in the palliative setting compared to GI (65%, 82/127) and breast trials (42%, 51/121) (*p* < 0.001). A total of 89% of trials evaluated systemic therapies (312/352), and 11% (40/352) tested radiotherapy, surgery, or a combination of modalities; this distribution differed substantially across disease sites (*p* = 0.009).

The majority of studies were superiority trials (84%, 297/352). Non-inferiority or equivalence trials were more common in studies of breast cancer (21%, 25/121) than GI (16%, 20/127) or lung cancer (10%, 10/104) (*p* = 0.075). OS was the primary endpoint in only 35% (122/352) of trials and was more likely to be the primary endpoint in lung (51%, 53/104) and GI cancer trials (43%, 55/127) than breast cancer trials (12%, 14/121) (*p* < 0.001). Surrogate primary endpoints were used in 55% (192/352) of trials; this was most common in breast cancer trials (72%, 87/121; GI (47%, 60/127), lung (43%, 45/104)) (*p* < 0.001).

### 3.3. Outcomes of RCTs

The results of clinical trials by disease site are shown in Table 2. The median number of participants across all trials was 494 (IQR 259–845). Breast cancer trials (666 patients, IQR 393–1505) were substantially larger than GI (438 patients, IQR 244–700) and lung trials (348 patients, IQR 212–627) (*p* < 0.001). Forty-five percent (160/352) of trials met their primary endpoint; this was more common in breast cancer (54%, 65/121) than GI (41%, 52/127) and lung cancer (41%, 43/104) (*p* = 0.024 breast cancer compared to GI/lung cancer combined).

Among superiority trials, 40% (118/297) were “positive” (*p* < 0.05 for the primary endpoint). Among positive superiority trials, the median effect size (hazard ratio) was 0.69 (IQR 0.65–0.75); this was comparable across disease sites (*p* = 0.137). An ESMO-MCBS grade could be calculated for 81 of the positive superiority trials; a total of 36% (29/81) met the threshold of substantial benefit. Lung cancer trials (50%, 15/30) were more likely to meet the threshold for substantial benefit than GI (30%, 7/23) or breast trials (25%, 7/28) (*p* = 0.041 lung trials compared to GI/breast trials combined).

### 3.4. Journal Impact Factors by Disease Site

**Figure 1:**
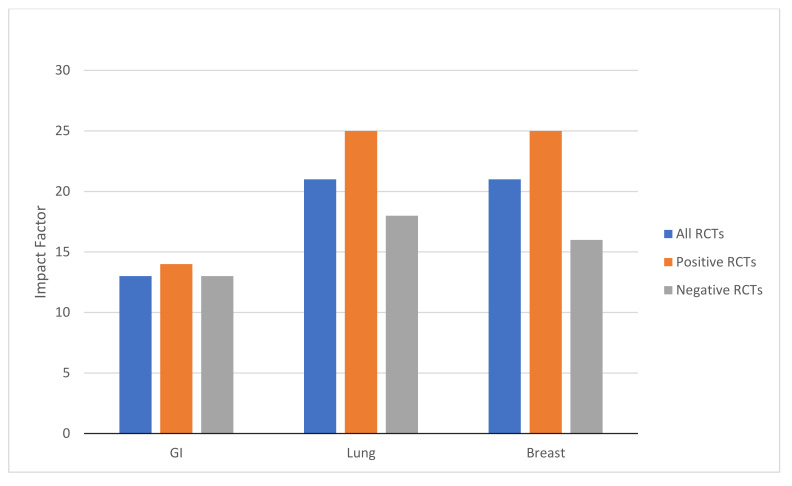
Differences in impact factors between GI (*n* = 126), lung (*n* = 103), and breast cancer (*n* = 119) randomized controlled trials (RCTs), stratified by positive and negative studies.

Ninety-nine percent (348/352) of RCTs were published in journals with an IF. The median IF for all RCTs was 18 (IQR 7–27). Positive trials (median IF 24, IQR 7–36) were published in higher-impact journals compared to negative trials (median IF 14, IQR 7–25, *p* = 0.002). GI trials were published in journals with a substantially lower IF compared to lung cancer and breast cancer trials (*p* = 0.038 GI trials compared to breast/lung trials combined) (Figure 1). GI trials (*n* = 126) were published in journals with a median IF of 13 (IQR 7–25), and there was no difference in IF between positive and negative studies (median IF 14 (IQR 7–27) vs. IF 13 (IQR 7–25), respectively, *p* = 0.489). Lung RCTs (*n* = 103) were published in journals with a median IF of 21 (IQR 7–34). Positive lung cancer trials were published in higher-impact journals (median IF 25 (IQR 9–53) vs. 18 (IQR 6–26), *p* = 0.024). Breast cancer trials (*n* = 119) were also published in journals with a median IF of 21 (IQR 7–34). Positive breast cancer RCTs were published in higher-impact journals (median IF 25 (IQR 7–36) vs. 16 (IQR 6–26), *p* = 0.019).

## 4. Discussion

This study provides an overview of the methodologic design and results of contemporary RCTs in GI, lung, and breast cancers. Several important findings were observed. First, these three diseases comprise 50% of all published RCTs in cancer. Second, 75% of RCTs in these cancers are funded by industry, and this was more common in breast and lung cancer trials compared to GI trials. Third, two thirds of all RCTs are conducted in the palliative setting, and only one third use OS as a primary endpoint. Fourth, RCTs of breast cancer are larger and considerably more likely to use surrogate endpoints than trials of lung and GI cancer. Fifth, half of lung cancer trials use OS as a primary endpoint, and when positive, these trials are more likely than breast and GI cancer trials to meet the ESMO-MCBS threshold for substantial clinical benefit. Finally, RCTs of GI cancer are less likely to be funded by industry and are published in substantially lower impact journals than studies of breast and lung cancer.

It is notable that only one third of RCTs have OS as a primary endpoint, and only a handful of trials use quality of life even in the palliative context. Moreover, use of OS varies across disease sites, with half of lung cancer trials using OS compared to only 12% of breast cancer trials. It is worth highlighting that there are substantial problems in using endpoints such as progression-free survival (PFS), which are very often not valid surrogates for OS [20]. Several factors may explain the observation that RCTs in breast cancer are more likely than RCTs in other cancers to use surrogate endpoints. These include the fact survival events often take longer to accrue in breast cancer and the reality that surrogate endpoints (i.e., pathological complete response (pCR) and event-free survival (EFS)) are accepted by regulatory agencies for breast cancer but not for other cancer types. However, despite their widespread use, a systematic review of all surrogate endpoints in breast cancer identified that pCR, DFS, relapse rate (RR), and PFS have only a weak to moderate correlation with OS; this limits their validity [21]. Notable exceptions include DFS in HER2-positive breast cancer which had a strong correlation, and EFS which had a moderate but not significant correlation with OS [22]. Conversely, in lung cancer, DFS is a strong surrogate for OS in older trials of cytotoxic chemotherapy and radiotherapy; it remains unknown whether this extends to trials of targeted/immune therapies [23]. Although lung cancer was more likely to use overall survival in our cohort than breast and GI cancer, other work suggests lung trials are also shifting towards surrogate endpoints [8]. The current US Food and Drug Administration Surrogate Endpoint Table lists PFS (for non-small cell lung cancer; NSCLC) and DFS (for adjuvant-stage III NSCLC) as surrogate measures that may be used in regulatory approval [24]. With regard to GI trials, since the prognosis has historically been poor and most treatment changes have been made on the basis of OS gains, the lower frequency of use of surrogate endpoints is not surprising. Accordingly, the FDA Table includes only ORR as a surrogate for gastroesophageal cancers, and ORR, PFS, and DFS as acceptable surrogates for colorectal cancer. Cross-over and subsequent lines of therapy may limit the ability of some RCTs to identify large differences in OS. Moreover, beyond surrogacy for OS, it remains unknown whether DFS/PFS has intrinsic meaning to patients.

With rising sample sizes, modern RCTs in cancer are also plagued by the tension between statistical significance and clinical significance [25,26]. Prior work has shown that the magnitude of benefit in contemporary clinical trials is decreasing over time [7,9]. The ESMO-MCBS provides an objective measure of the extent to which new cancer therapies offer benefit to patients. We have previously applied the ESMO-MBCS to a cohort of 277 published RCTs in breast, colorectal, and NSCLC and found that only 43%, 28%, and 20% of “positive” trials, respectively, demonstrated substantial clinical benefit [27]. The results in the current study are consistent with this for breast and GI cancers; however, we found that a greater proportion of trials in lung cancer met the ESMO-MCBS threshold for substantial benefit. This may be related to the large improvements seen with targeted therapy and immunotherapy in trials published during the study period [28,29,30,31].

Our findings related to the growing role of industry in funding cancer clinical trials are consistent with our prior work. We have previously described that the proportion of breast, colorectal, and non-small cell lung cancer systemic therapy trials funded by industry increased from 4% in 1975–1984 to 78% in 2005–2009 [8]. Historically, a larger proportion of RCTs were funded by government grants [7]. The reasons for this substantial shift in RCT funding are multifactorial and likely reflect a decreased availability of government grants, high costs of cancer medicines incentivizing industry, and the increased complexity and expense associated with drug development and conducting contemporary RCTs [32,33].

Finally, our results highlight the persistent problem in oncology of publication bias [34,35]. This bias is illustrated by the fact that negative RCTs are published in journals with much lower IFs. It is also seen between cancer types; GI trials were published in lower-impact journals. Notably, positive GI RCTs were still published in much lower IF journals than negative trials of breast cancer. We have previously described the influence of media and government on cancer research funding and publications; breast cancer has a disproportionate volume of research output relative to its global mortality burden [36,37].

Our study findings should be considered in light of some methodologic limitations. First, the GI cohort included multiple cancer sites, and lung cancer trials included both non-small cell lung cancer and small cell lung cancer. Thus, it is not clear if the study results apply to all types of GI and lung cancer. Second, our study is limited to English-language phase III RCTs and, therefore, does not reflect the entire cancer research ecosystem. Finally, the landscape of oncology continues to evolve rapidly, and as such, our results do not take into account pivotal RCTs of immunotherapy published in recent years.

## 5. Conclusions

In summary, we have observed important differences in RCT design and output between the three major cancers. Use of surrogate endpoints and the magnitude of benefit associated with new treatments vary substantially across cancer sites. The current balance of the cancer research ecosystem may need to be re-calibrated given the overwhelming focus on studies of new cancer medicines in the palliative context. Finally, cancer clinical trials are often designed by site-specific research groups. These comparative data offer insights to allow for improved design and interpretation of future RCTs across the major cancer sites.

## Figures and Tables

**Table 1:** Design characteristics of phase III RCTs in gastrointestinal, lung, and breast cancer published globally between 2014 and 2017.

	All RCTs	GI	Lung	Breast	*p-* Value
	*n* = 352	*n* = 127	*n* = 104	*n* = 121	
	*n* (%)				
Treatment intent					
Palliative	223 (63)	82 (65)	90 (87)	51 (42)	<0.001
Curative	14 (4)	7 (6)	3 (3)	4 (3)	
Neoadjuvant/adjuvant	115 (33)	38 (30)	11 (11)	66 (55)	
					
Experimental arm					
Systemic	312 (89)	110 (87)	91 (88)	111 (92)	0.009
Radiation	18 (5)	4 (3)	6 (6)	8 (7)	
Surgery	9 (3)	7 (6)	0 (0)	2 (2)	
Other/Combinations	13 (4)	6 (5)	7 (7)	0 (0)	
					
Study design					
Superiority	297 (84)	107 (84)	94 (90)	96 (79)	0.075
NI/equivalence	55 (16)	20 (16)	10 (10)	25 (21)	
					
Primary endpoint					
OS	122 (35)	55 (43)	53 (51)	14 (12)	<0.001
DFS/EFS/RFS	84 (24)	28 (22)	7 (7)	49 (40)	
PFS/TTF	108 (31)	32 (25)	38 (37)	38 (31)	
Other	38 (11)	12 (9) *	6 (6) ^#^	20 (17) ^^^	
					
Country of origin ^@^					
HIC	315 (89)	116 (91)	84 (81)	115 (95)	0.002
LMIC	37 (11)	11 (9)	20 (19)	6 (5)	
					
Industry funding ^~^	263 (75)	85 (67)	82 (79)	96 (79)	0.037

RCTs = randomized controlled trials; GI = gastrointestinal; OS = overall survival; DFS = disease-free survival; EFS = event-free survival; RFS = relapse-free survival; PFS = progression-free survival; TTF = time to treatment failure; QOL = quality of life; NI = non-inferiority; HIC = high-income country; LMIC = low-middle-income country. * GI—other primary endpoints include QOL/toxicity (*n* = 3), response rate (*n* = 6), and other (*n* = 3). ^#^ Lung—other primary endpoints include QOL/toxicity (*n* = 3) and response rate (*n* = 3). ^ Breast—other primary endpoints include QOL/toxicity (*n* = 3), response rate (*n* = 10), and other (*n* = 7). ^@^ Based on institutional affiliation of first author. ^~^ Funding was unstated for *n* = 10 RCTs (4 breast, 3 lung, 3 GI).

**Table 2:** Results of RCTs in gastrointestinal, lung, and breast cancer published globally between 2014 and 2017.

	All RCTs	GI	Lung	Breast	*p*-Value
	*n* = 352	*n* = 127	*n* = 104	*n* = 121	
	*n* (%)				
Total sample size					
Median (IQR)	494 (259–845)	438 (244–700)	348 (212–627)	666 (393–1505)	<0.001
					
Primary endpoint met					
Yes	160 (45)	52 (41)	43 (41)	65 (54)	0.079
No	192 (55)	75 (59)	61 (59)	56 (46)	
					
HR for + RCTs	*n* = 118 *	*n* = 38 ^a^	*n* = 36 ^b^	*n* = 44 ^c^	
Median (IQR)	0.69 (0.65–0.75)	0.71 (0.66–0.75)	0.67 (0.62–0.72)	0.69 (0.67–0.75)	0.137
					
ESMO-MCBS grade	*n* = 81	*n* = 23	*n* = 30	*n* = 28	
Substantial benefit (A, B, 4, 5)	29 (36)	7 (30)	15 (50)	7 (25)	0.114
Not substantial benefit (C,1,2,3)	52 (64)	16 (70)	15 (50)	21 (75)	

RCT = randomized controlled trial; GI = gastrointestinal; IQR = interquartile range; HR = hazard ratio; ESMO-MCBS = European Society of Medical Oncology-Magnitude of Clinical Benefit Scale. * Only reported for *n* = 104 positive superiority trials; HR unavailable for 14. ^a^ Only reported for *n* = 38 positive superiority trials; HR unavailable for 3. ^b^ Only reported for *n* = 36 positive superiority trials; HR unavailable for 2. ^c^ Only reported for *n* = 44 positive superiority trials; HR not reported for 9.

## Data Availability

All data can be found in the text.
